# Supplementary material for: Atomic‐Scale Studies of Fe3O4(001) and TiO2(110) Surfaces Following Immersion in CO2‐Acidified Water
Source: Chemphyschem. 2020 Aug 3;21(16):1788–96. doi: 10.1002/cphc.202000471 (PMC7522689; doi:10.1002/cphc.202000471)
Supplement: Supplementary file 1 — Supplementary [file CPHC-21-1788-s001.pdf]

ChemPhysChem

Supporting Information

Atomic-Scale Studies of Fe_3O_4(001) and TiO_2(110) Surfaces Following Immersion in CO_2-Acidified Water

Francesca Mirabella, Jan Balajka, Jiri Pavelec, Markus Göbel, Florian Kraushofer, Michael Schmid, Gareth S. Parkinson, and Ulrike Diebold*

Supplemental Information

Equilibria involved in the CO_2 dissolution in water, acidic constants and pH values.

CO_2 dissolves in liquid water and then forms H_2CO_3 according to the equilibria (1) and (2) respectively:

(1) $CO_{2(g)} \leftrightarrow CO_{2(aq)}$

(2) $CO_{2(aq)} + H_2O \leftrightarrow H_2CO_3$ $\qquad K_r = \frac{[H_2CO_3]}{[CO_{2(aq)}]} \approx 1.7 \cdot 10^{-3}$

The reaction constant K_r [1] of equation (2) indicates that the forward reaction is slow with respect to carbonic acid decomposition [2]. At equilibrium, only a small fraction of the dissolved CO_2 is converted into carbonic acid, while most of the CO_2 remains as molecular CO_2.

Carbonic acid is a weak acid, which dissociates in two steps to bicarbonate (HCO_3^-) and carbonate (CO_3^{2-}), respectively, as described by the equilibria (3) and (4):

(3) $H_2CO_3 + H_2O \leftrightarrow HCO_3^- + H_3O^+$ $\qquad K_{a1} = 2.5 \cdot 10^{-4}$

(4) $HCO_3^- + H_2O \leftrightarrow CO_3^{2-} + H_3O^+$ $\qquad K_{a2} = 4.7 \cdot 10^{-11}$

A weak acid only partially dissociates into its ions in aqueous solutions, and the corresponding acidic constant can be considered as a quantitative measure of the strength of an acidic solution. In the case of carbonic acid, the two acidic constants [3], related to the two dissociation steps, are indicated in (3) and (4) as K_{a1} and K_{a2}. Given that K_{a2} << 1, we can assume that the concentration of CO_3^{2-} is always negligible compared to HCO_3^-. Consequently, the equilibrium (4) plays no quantitative role in the pH of the solution. Considering, then, only the sum of the equilibria (2) and (3), we will have a new equilibrium (5) with the corresponding acidic constant necessary for the pH calculation of our solution (6), as described below:

(5) $CO_{2(aq)} + 2H_2O \leftrightarrow HCO_3^- + H_3O^+$

(6) $K_A = K_r \cdot K_{a1} = 4.2 \cdot 10^{-7}$

By increasing the partial pressure of CO_2 surrounding the water drop, we are able to shift the described equilibria more to the right, resulting in the acidification of the water. We can use Henry´s law [4] (equation (7)), which describes the equilibrium between the partial pressure of a gas above the liquid and the concentration of this gas dissolved in the liquid, to tune the pH of the water drop to the desired value:

(7) $H^{pc} = {p_{CO_2}}/{[CO_{2(aq)}]}$

where H^{pc} is Henry´s constant ($29.4 \cdot 10^3\ {mbar \cdot \mathrm{L}}/{mol}$ at $25\ °C$), p_{CO_2} is the partial pressure of the CO_2, and $[CO_{2(aq)}]$ is the concentration of the species in the aqueous phase.

The pH can be written as a function of the CO_2 concentration as shown in equation (8):

(8) $pH_{(p)} = -\,log\sqrt{[H_3O^+]} = -\,log\sqrt{K_A \cdot [CO_{2(aq)}]} = -\,log\sqrt{K_A\ \left({p_{CO_2}}/{H^{pc}}\right)}$

Using this approach, by equilibrating our water drop with 20 mbar, 800 mbar, and 1 bar of CO_2, we create acidic solutions with pH values of 4.8, 4.0, and 3.9 respectively.

Table 1 shows the concentration values calculated using the equations described above, for the dissolved species in water as a function of the p_{CO_2} surrounding the water drop. While the bicarbonate concentration increases as the pH decreases, the carbonate concentration can be considered constant around the value of 5.6×10^{-11} mol/L.

p_{CO_2} (mbar)	pH	$[CO_2]$ (mol/L)	$[H_2CO_3]$ (mol/L)	$[HCO_3^-]$ (mol/L)	$[CO_3^{2-}]$ (mol/L)
20	4.8	$6.8 \cdot 10^{-4}$	$1.2 \cdot 10^{-6}$	$1.7 \cdot 10^{-5}$	$5.6 \cdot 10^{-11}$
200	4.3	$6.8 \cdot 10^{-3}$	$1.2 \cdot 10^{-5}$	$5.3 \cdot 10^{-5}$	$5.6 \cdot 10^{-11}$
800	4	$2.7 \cdot 10^{-2}$	$4.6 \cdot 10^{-5}$	$1.1 \cdot 10^{-4}$	$5.6 \cdot 10^{-11}$
1000	3.9	$3.4 \cdot 10^{-2}$	$5.8 \cdot 10^{-5}$	$1.2 \cdot 10^{-4}$	$5.6 \cdot 10^{-11}$

Table 1. Dissolved species concentrations calculated as a function of p_{CO_2} surrounding the water drop.

Figure S1.

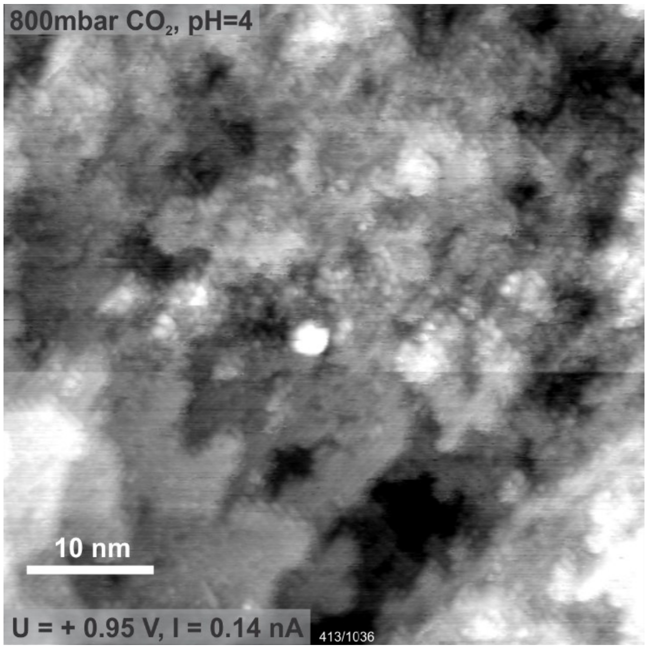

S1. ***STM image taken at room temperature of the Fe_3O_4(001) surface after exposing to acidic solution at pH 4.0.***

Figure S2.

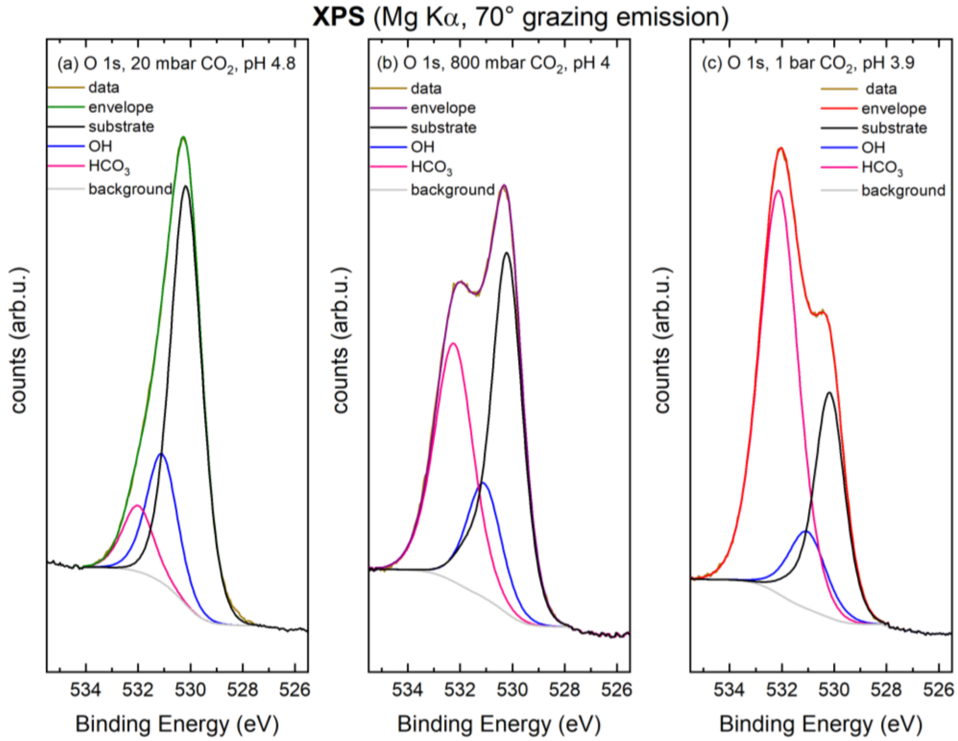

S2. ***O 1s XPS spectra measured after exposing the Fe_3O_4(001) surface to acidic solutions at (a) pH 4.8, (b) 4.0, and (c) 3.9, and corresponding peak fits.*** *The peak fits as well as their integral area were obtained using the software CasaXPS.*

The XPS O 1s region after exposing the Fe_3O_4(001)-(√2 × √2)R45°surface to acidic solutions at (a) pH 4.8, (b) 4, and (c) 3.9 is shown in Fig. S2. The contributions from the surface oxygen (black) as well as from the surface OH group (blue) decrease as the pH decreases, while the bicarbonate contribution (pink) increases. At pH 4.8, the surface OH corresponds to 19.9% of the overall O 1s area, and decreases to 15.9% and to 11.1%, at pH 4 and 3.9, respectively. The HCO_3^- contribution corresponds to the 10.2% of the overall O 1s area when the pH of the acidic solution is 4.8, and increases to 36.6% and to 61.1%, as the pH decreases to 4 and to 3.9, respectively. These results follow the same trend of the surface bicarbonate concentrations measured based on the C 1s integral area reported in Fig. 3.

References

[1] C.E Housecroft and A. G. Sharpe, Inorganic Chemistry, 2nd Edition *Pearson* **2005**, 579.

[2] E. Magid, B. O. Turbeck, *Biochim. Biophys. Acta* **1968**, 165, 515–524.

[3] N. Greenwood, A. Earnshaw, Chemistry of Elements, 2nd Edition, *Butterworth Heinemann* **1997**.

[4] R. Sander, *Atmos. Chem. Phys.* **2015**, 4399–4981.
